# AI-ChatGPT/GPT-4: An Booster for the Development of Physical Medicine and Rehabilitation in the New Era!

**DOI:** 10.1007/s10439-023-03314-x

**Published:** 2023-07-27

**Authors:** Shengxin Peng, Deqiang Wang, Yuanhao Liang, Wenshan Xiao, Yixiang Zhang, Lei Liu

**Affiliations:** 1 School of Rehabilitation Medicine of Binzhou Medical University, Yantai, China; 2 Weifang Medical University, Weifang, China; 3 Shandong First Medical University, Jinan, China; 4 Department of Painology, The First Affiliated Hospital of Shandong First Medical University (Shandong Provincial Qianfoshan Hospital), Jinan, 250014 China

**Keywords:** Artificial intelligence, ChatGPT, GPT-4, Physical medicine and rehabilitation

## Abstract

Artificial intelligence (AI) has been driving the continuous development of the Physical Medicine and Rehabilitation (PM&R) fields. The latest release of ChatGPT/GPT-4 has shown us that AI can potentially transform the healthcare industry. In this study, we propose various ways in which ChatGPT/GPT-4 can display its talents in the field of PM&R in future. ChatGPT/GPT-4 is an essential tool for Physiatrists in the new era.

## Introduction

In recent years, advances in AI have been transforming the healthcare industry. The analysis of medical images, the detection of drug interactions, the identification of high-risk patients, and the coding of medical notes are just a few of the many uses of AI in medicine [1]. The recent release of ChatGPT by OpenAI has sparked an enormous response in the academic community [2, 3], with several top journals, including Nature, Science, and the New England Journal of Medicine, discussing its impact on medicine [4–7].

ChatGPT is a language model based on the transformer architecture [4], and after a large amount of text data training, it has produced human-like language text that allows users to receive information intuitively [8]. Moreover, the most surprising feature is its chat function and powerful ability to search massive amounts of information, provide professional answers, make reasonable suggestions, and efficiently process and integrate data [5, 9].

In more than five days of open beta, ChatGPT surpassed one million registered users and 100 million monthly active users as of January 2023 [1, 6]. It is worth noting that OpenAI has released the latest version of GPT-4, which has significantly improved over the previous GPT-3.5 version in terms of increased map reading capability, answer accuracy, and text input restrictions [1, 6]. GPT-4 has exhibited a performance comparable to that of human individuals across professional and academic benchmarks. Notably, it has excelled in the mock bar exam, surpassing the scores of 90% of test takers, and has achieved commendable results on the US medical licensing exam [10]. Although studies have explored the role of ChatGPT/GPT-4 in radiology, sports medicine, obstetrics and gynecology, infectious diseases, neurosurgery, spinal surgery, and various other medical disciplines [11–16], to date, no studies have analyzed the potential of ChatGPT/GPT-4 in multiple applications in PM&R.

Physical Medicine and Rehabilitation, as one of the four major medical disciplines juxtaposed with health care medicine, preventive medicine, and clinical medicine, are indispensable core parts in the development and balance of the human medical and health system. Physical Medicine and Rehabilitation (PM&R), also known as Physiatry, helps patients regain control of their lives by restoring function [17]. This area focuses on the ability of persons to perform their best positions within the limits of the process that may not cure the disease. The point is to try as far as possible to fully recover to the level of pre-illness function or to optimize the quality of life of those who may not fully recover. With the establishment of the American Board of Physical Medicine and Rehabilitation (ABPMR) in 1947, PM&R was officially recognized as a profession by the American Medical Association (AMA) [18].

PM&R is a relatively new global field of medicine that continues to evolve to meet the medical needs of patients with the fundamental goal of improving functional performance and enhancing the quality of life. Among the many awards established by the American Academy of Physical Medicine and Rehabilitation Medicine (AAPMR), the Frank H. Krusen Award is considered among the most prestigious honors. Notably, the award was presented to Henry Betts and Joel Press, founders of the Center for Spine and Sports Rehabilitation at the Rehabilitation Institute of Chicago, who emphasized that "medicine adds years to people's life, Physiatry adds quality of those years. [17]”

"Professors Joe and De Lisa have pointed out that introducing and implementing new technologies are essential to improve rehabilitation care. In future, information, primary and allied Physiatrists will be at the heart of the development of rehabilitation [19]. With the advent of ChatGPT/GPT-4, a revolution has begun. Given this, our group conducted an online survey on this subject. In this study, we focus on describing four leading potential roles of ChatGPT/GPT-4 for the field of PM&R.

## The Poverty of Rehabilitation Physicians Crisis

The shortage of rehabilitation physicians is a global problem. According to 2009 data, 16,000 physicians, 14,000 therapists, and 12,000 nurses worked in medical rehabilitation in China. However, data as of 2018 show that there are 38,260 physicians and 15,514 nurses in rehabilitation hospitals. It is estimated that approximately 40,000 therapists are practicing. The demand for rehabilitation physicians, therapists, and nurses is expected to increase to approximately 60,000, 150,000, and 60,000, respectively, within ten years [20].

In Japan, the demand for rehabilitation physicians is also rapidly increasing. The total number of rehabilitation physicians in Japan increased by 175% between 1996 and 2016, but it is mainly concentrated in physicians aged 40 years and above [21]: policy initiatives and the rapidly increasing demand for rehabilitation due to an aging society. Similarly, there is a national shortage of PRM physicians in the United States, and PRM faces severe challenges in recruiting trainees [22].

The shortage of rehabilitation physicians is a global problem, but society's rehabilitation problems are increasing. The emergence of ChatGPT/GPT-4 may find a solution between the poverty of rehabilitation physicians and the heavy clinical load. Isaac Kohane is a physician and chair of the Biomedical Informatics Program at Harvard Medical School. He had the opportunity to test GPT-4 last fall and was so impressed that he quickly turned it into a book called "The AI Revolution in Medicine: GPT-4 and Beyond," which is already available on Amazon [23]. He has said that one of the most significant benefits of AI is to help reduce or eliminate tedious paperwork time that is now keeping physicians from spending enough time with their patients, which often leads to burnout.

## Medical History Taking and Triage

ChatGPT/GPT-4 offers an interactive dialogue interface, efficiently acquiring a large volume of patient complaints and symptoms while effectively organizing and structuring the collected information to ensure the accurate recording of essential details. It facilitates the identification of pertinent information and potential risk factors and provides recommendations for additional inquiries. Through comprehensive analysis and summarization of patient data, it supports healthcare professionals in conducting thorough medical history collection, streamlining the process of patient information gathering, and enhancing the understanding of their medical background.

Moreover, ChatGPT/GPT-4 assists in the triage process by analyzing symptoms, vital signs, and other patient data. It provides initial assessments of urgency and severity, aiding healthcare professionals in prioritizing patients based on the provided information and offering preliminary guidance to determine the appropriate level of care or intervention required. This capability contributes to the efficient allocation of healthcare resources.

## Rehabilitation Assessment

Rehabilitation assessment uses objective and quantitative methods to effectively and accurately evaluate the nature, type, scope, severity, and prognosis of a patient's functional impairment. Rehabilitation assessment is an integral part of PM&R. Without proper assessment, it is impossible to formulate an effective treatment plan and evaluate the effect of rehabilitation treatment. ChatGPT/GPT-4 has potential applications in rehabilitation assessment. First, ChatGPT/GPT can learn a lot of rehabilitation assessment data in depth, extract useful information, and generate statistical analysis reports through data analysis and pattern recognition. Secondly, ChatGPT/GPT-4 can develop personalized rehabilitation assessment tools, providing customized questionnaires, scales, or tests based on patient characteristics and needs. In addition, ChatGPT/GPT-4 can also perform data mining and prediction to uncover correlations and trends in rehabilitation assessment data and predict difficulties and progress in patients' rehabilitation process. Finally, ChatGPT/GPT-4 can automatically generate detailed assessment reports and integrate various assessment data to improve work efficiency and accuracy.

## Scientific Research

ChatGPT/GPT-4 has a wide range of potential applications in various fields of scientific research [24, 25], and it can provide researchers with comprehensive assistance. First, ChatGPT/GPT-4 can assist in literature reviews, helping researchers to retrieve and understand the latest scientific advances. Second, it can be used for data analysis and interpretation, assist in experimental design, and provide a summary and performance of experimental results. ChatGPT/GPT-4 can participate in discussions and brainstorming as a virtual collaborator, giving new perspectives and questions. Previous studies have demonstrated the accuracy of ChatGPT/GPT-4 in generating systematic evaluation ideas in the field of plastic surgery [26].

Furthermore, among scientists whose native language is not English, ChatGPT/GPT-4 has been of great help in translation and retouching, and it can even retouch manuscripts according to the requirements of different journals. However, it is essential to note that the data collection process of ChatGPT/GPT-4 may inadvertently increase the risk of plagiarism. In conclusion, ChatGPT/GPT-4 is a valuable tool in physical medicine and rehabilitation research, providing comprehensive support and guidance for researchers.

## Rehabilitation Education

Rehabilitation education plays a vital role in PM&R and is designed to provide patients, families, and healthcare professionals with knowledge and skills about the rehabilitation process, self-management, and rehabilitation support. The goal is to help patients and their families understand the effects of the illness or injury, learn self-care skills for the rehabilitation process, and promote positive recovery outcomes. The importance of rehabilitation education is reflected in several ways. First, ChatGPT/GPT-4 serves as a virtual rehabilitation education partner that provides patients and families with extensive information and answers to their questions about the rehabilitation process, self-management skills, and rehabilitation goals, thereby increasing their awareness of rehabilitation. By interacting with ChatGPT/GPT-4, patients and families can learn about the concepts, goals, and benefits of rehabilitation and better understand the challenges and expectations of the rehabilitation process, leading to increased awareness and participation in rehabilitation. At the same time, ChatGPT/GPT-4 can facilitate patient and family self-management and rehabilitation by providing education and guidance on disease management, medication management, daily living skills, and self-monitoring. By learning effective self-management strategies provided by ChatGPT/GPT-4, patients and family members can actively participate in rehabilitation and improve their recovery and quality of life. In addition, ChatGPT/GPT-4 can help healthcare professionals enhance their communication and education skills better to meet the educational needs of patients and families. It can be a communication platform to help healthcare professionals explain diagnoses and treatment plans, answer questions, and provide support and guidance. Finally, as a virtual rehabilitation education partner, ChatGPT/GPT-4 can interact with patients and provide support and monitoring during rehabilitation. Through regular communication, ChatGPT/GPT-4 can understand the patient's rehabilitation progress, confusion, or needs and provide rehabilitation education support accordingly. It can serve as an ongoing partner to help patients maintain recovery motivation, actively participate in the rehabilitation process, and enhance the effectiveness of recovery.

## Limitations

While ChatGPT/GPT-4 has a wide range of applications in various fields, it currently has some limitations in the healthcare industry, including tendencies to “hallucinate” incorrect information, exhibit problematic social biases, and misbehave or assume disturbing personas when given an “adversarial” prompt. Although the incidence of hallucinations has gradually decreased with each iteration, the medical community still needs to identify the information provided by ChatGPT/GPT-4 carefully. Another concern is that the privacy and security of using medical information as an emerging AI model have yet to be validated, and legislation has yet to be improved. Data regulators around the world are investigating how OpenAI collects data used to train its large language models, the accuracy of the answers it provides about people and other legal issues with the use of its text generation system. In Europe, General Data Protection (GDPR) laws require companies and organizations to prove a legitimate reason for processing people's personal information, allow people to access information about them, understand how their information is used, and correct errors.

In some cases, they can request that certain data types be deleted. How personal information is used in training data has been an early concern for EU regulators. OpenAI says its large language model is trained on three sources of information: (1) information that is publicly available on the Internet, (2) information that we license from third parties, and (3) information that our users or human trainers provide. This can include information about individuals. "A large amount of data on the Internet relates to people, so our training information does incidentally include personal information,” OpenAI noted, adding that it is taking steps to reduce the handling of personal information. In short, there is a pressing need to prevent the leakage and misuse of patient information.

## Conclusion

This study explored several application aspects of ChatGPT/GPT-4 in the field of PM&R, such as history taking and triage, rehabilitation assessment, scientific research, and rehabilitation education. The promotion of these applications will greatly promote the development and innovation of PM&R. We believe that the future of PM&R will involve a great deal of AI-related research and that AI-based tools such as ChatGPT/GPT-4 will play an important role.

However, the development of AI is a continuous process of progress. While we enjoy the benefits of AI, there are issues to be aware of, such as data security and privacy, ethical and moral issues, and the risk of misuse and misappropriation. As we continue to promote the application and research of AI, we need to proactively address these issues and develop reasonable norms and guidelines to guide the development of AI. By addressing these issues, PM&R can take full advantage of the power of AI and continue to advance to provide better quality rehabilitation services and improve patients' rehabilitation outcomes and quality of life.

## Data Availability

Not applicable.
